# Individual and community level factors associated with discriminatory attitudes against people living with HIV/AIDS among women of reproductive age in three sub-Saharan African countries: evidence from the most recent demographic and health survey (2021/22)

**DOI:** 10.1186/s12889-024-19022-7

**Published:** 2024-06-05

**Authors:** Belayneh Shetie Workneh, Alebachew Ferede Zegeye, Tadesse Tarik Tamir, Mohammed Seid Ali, Temesgen Ayenew, Enyew Getaneh Mekonen

**Affiliations:** 1 Department of Emergency and Critical Care Nursing, School of Nursing, College of Medicine and Health Sciences, University of Gondar, Gondar, Ethiopia; 2 Department of Medical Nursing, School of Nursing, College of Medicine and Health Sciences, University of Gondar, Gondar, Ethiopia; 3 Department of Pediatrics and Child Health Nursing, School of Nursing, College of Medicine and Health Sciences, University of Gondar, Gondar, Ethiopia; 4 Department of Emergency and critical care nursing, College of health sciences, Debremarkos Markos University, Debre Markos, Amhara, Ethiopia; 5 Department of Surgical Nursing, School of Nursing, College of Medicine and Health Sciences, University of Gondar, Gondar, Ethiopia

**Keywords:** Africa, Women, HIV/AIDS, Sub-Saharan, Discriminatory attitudes

## Abstract

**Introduction:**

HIV-related stigma and discrimination significantly affects health, and well-being, willingness to be tested for HIV, initiation and adherence to antiretroviral therapy, and quality of life. However, the findings of the prior studies revealed that the prevalence of discrimination against people living with HIV is high. Thus, we aimed to assess the magnitude of discriminatory attitudes against people living with HIV/AIDS and associated factors in three sub-Saharan African countries.

**Methods:**

The appended and most recent Demographic and Health Survey dataset of three sub-Saharan African countries from 2021 to 2022 was used for data analysis. A total of 56,690 women aged 15–49 years were included in this study as a weighted sample. The determinants of discriminatory attitudes against people living with HIV/AIDS were determined using a multilevel mixed-effects logistic regression model. Significant factors associated with discriminatory attitudes against people living with HIV/AIDS in the multilevel mixed-effect logistic regression model were declared significant at *p*-values < 0.05. The adjusted odds ratio (AOR) and confidence interval (CI) were used to interpret the results.

**Result:**

The overall prevalence of discriminatory attitudes against people living with HIV/AIDS was 28.19% (95% CI: 27.74%, 28.64%). In the multivariable analysis, individual level (being young, being an internet user, being tested for HIV, and having comprehensive knowledge about HIV) and community level (being a rural dweller) were factors associated with discriminatory attitudes against people living with HIV/AIDS.

**Conclusion:**

The prevalence of discriminatory attitudes against people living with HIV/AIDS in three sub-Saharan African countries was high. Individual and community-level variables were associated with discriminatory attitudes against people living with HIV/AIDS. Therefore, special consideration should be given to rural dwellers and young adults. In addition, better to strengthen the accessibility of Internet and HIV testing services, and improve HIV-related education to reduce the magnitude of discriminatory attitudes against people living with HIV/AIDS.

**Supplementary Information:**

The online version contains supplementary material available at 10.1186/s12889-024-19022-7.

## Introduction

Since the beginning of the epidemic, about 85.6 million people have been infected with HIV, and about 40.4 million people died worldwide [1]. The burden of the global HIV epidemic is disproportionately concentrated in sub-Saharan Africa, where in 2017, about 75% of deaths and 65% of new infections occurred and 71% of people living with HIV resided [2]. The world community calls repeatedly for the end of the HIV epidemic. Sustainable Development Goal 3 (ensure healthy lives and promote well-being for all at all ages) calls for the end of the HIV epidemic by 2030. The Joint United Nations Programme on HIV/AIDS (UNAIDS) fast-track strategy also targets the diagnosis and treatment of HIV aimed to lower the number of new infections and deaths by the year 2030. Despite these objectives, a recent assessment of the HIV situation found that the global HIV epidemic is not expected to end [3–6].

Important factors like stigma and discriminatory attitudes should be considered for effective HIV programming. Stigma refers to negative feelings, attitudes, beliefs, and perceptions about individuals living with HIV/ADIS, while discrimination is behaving differently against someone who is stigmatized [7–9]. Due to the nature of the disease contagiousness, transmissibility, incurability, fatality, transmission modality (sexually transmitted), and upsetting appearance of people living with advanced stage of HIV, they are prone to stigma and discrimination. HIV is primarily transmitted through unsafe sexual intercourse. Thus, it is widely viewed as a consequence of immoral behaviors which in turn results in stigmatizing and discriminating people living with HIV [10, 11].

HIV-related stigma and discrimination significantly affect the health, and well-being [12], willingness to be tested for HIV [13–17], initiation and adherence to antiretroviral therapy [15, 18–21], and quality of life [22, 23]. The findings of the prior studies revealed that the prevalence of discrimination against people living with HIV is 62.66% in Ethiopia [24], 47.08% in sub-Saharan Africa [25], 30.3% in Zambia [26], 42% in Botswana [27], and 50% in Saudi Arabia [28]. Studies have shown that being younger age, being a rural dweller, having no formal education, have no media exposure, male-headed household, not tested for HIV, lack of comprehensive knowledge about HIV, household wealth index, marital status, internet utilization status, and community level education [25, 29, 30], are factors associated with discriminatory attitudes against people living with HIV/ADIS.

Even though interventions to lessen discriminatory attitudes are necessary to prevent the spread of HIV/AIDS and improve the quality of life of people living with HIV, factors associated with discriminatory attitudes in sub-Saharan Africa are not well studied. Thus, our study aimed to assess the magnitude of discriminatory attitudes against people living with HIV/AIDS and associated factors in three sub-Saharan African countries. This could support policymakers and concerned bodies in their efforts to combat this grave issue at the national and local levels.

## Methods

### Study setting, period, design, and source

We have used the appended and most recent demographic and health survey (DHS) dataset of three sub-Saharan African countries, conducted in 2021/22, to assess discriminatory attitudes against people living with HIV/AIDS and its associated factors among women of reproductive age in the region with multilevel analysis. DHS is a community-based cross-sectional study conducted every five years to examine health and health-related indicators.

### Study population and sampling technique

The appended data from the most recent DHS surveys of three sub-Saharan African countries (Gabon, Kenya, and Tanzania) was used for data analysis to assess discriminatory attitudes against people living with HIV/AIDS and its associated factors among women of reproductive age. Different datasets, like those for men, women, children, births, and households, are included in the survey for each country. The DHS uses a stratified two-stage cluster design, with the first stage consisting of enumeration areas and the second stage generating a sample of households from each enumeration area. To determine the outcome variable (discriminatory attitudes against people living with HIV/AIDS), two discriminatory attitude questions, (I) children with HIV should be allowed to attend school with children without HIV (v857a) and (II) would you buy vegetables from vendors with HIV (v825) were used from the individual record. A binary logistic regression model was applied to determine the factors associated with the outcome variable and reported in terms of an adjusted odds ratio (AOR) with a significance level of 95%. In the univariable analysis, at 95% confidence intervals, a *p*-value of < 0.25 was considered a candidate for the multivariable analysis of the data. All variables with *p* values < 0.05 were considered statistically significant. A total weighted sample of 56,690 participants was included in the study (Table 1).

**Table 1:** Sample size for discriminatory attitudes against people living with HIV/AIDS among women of reproductive age in three sub-Saharan African countries

Country	Year of survey	Weighted sample(*n*)	Weighted sample (%)
Gabon	2021	9,400	16.58
Kenya	2022	32,036	56.51
Tanzania	2022	15,254	26.91
Total sample size		56,690	100.00

### Eligibility criteria

#### Inclusion

All women at the ages of reproductive life in three SSA countries were included.

#### Exclusion

Women who were not available in the selected enumeration areas were excluded from the analysis.

### Study variables

#### Dependent variable

The outcome variable of this study was discriminatory attitudes against people living with HIV/AIDS. To determine the outcome variable of the study, the two discriminatory attitudes questions “Children with HIV should be allowed to attend school with children without HIV (v857a) and Would you buy vegetables from vendors with HIV (v825)” were used. The woman was considered to have discriminatory attitudes against people living with HIV/AIDS if they answered “no” to both questions.

#### Independent variables

The independent variables of this study were individual level (age, education, having media exposure, wealth index, marital status, working status, distance to the health facility, husband education level, comprehensive knowledge about HIV/AIDS, testing for HIV, and internet utilization status) variables and community level (residence, community media exposure, and community illiteracy level) variables.

### Data management and statistical analysis

The most recent DHS data of 3 sub-Saharan African countries (Gabon, Kenya, and Tanzania) was downloaded from the Demographic and Health Survey (DHS) program website and appended to have a single data set. Missing values have been managed by dropping variables assigned as don’t know and unspecified numbers. The cleaned and recorded data were analyzed using STATA (version 14) statistical software. The DHS data variables are organized in clusters, and those in a cluster are more similar to one another than those of other clusters. The assumptions of independent observations and equal variance across clusters were violated to use a standard logistic regression model. This implies that using a sophisticated model to take into account cluster factors is necessary. Given this, multilevel mixed-effects logistic regression was used to determine the factors associated with discriminatory attitudes against people living with HIV/AIDS. Four models were used in multilevel mixed effect logistic regression: model I (the association of individual-level variables with the outcome variable), model II (the association of community-level variables with the outcome variable), model III (the association of both individual and community-level variables with the outcome variable), and the null model (outcome variable only). The association of both individual and community-level variables was fitted simultaneously in the final model (model III).

### Model building

The deviance and log-likelihood tests were used to compare the models, and the model with the lowest deviance and the highest log-likelihood ratio was selected as the best-fitted model. Moreover, multicollinearity was tested using the variance inflation factor (VIF), and a VIF value of less than five for each independent variable with a mean VIF of 1.97, indicating there was no significant multicollinearity between independent variables.

### Random effects

The random effects or measures of variation of the outcome variable were estimated by the median odds ratio (MOR), intra-class correlation coefficient (ICC), and proportional change in variance (PCV). The proportional change in variance (PCV) and intra-class correlation coefficient (ICC) were calculated to measure the variation between clusters. Taking clusters as a random variable, the ICC reveals the variation of discriminatory attitudes against people living with HIV/AIDS between clusters, which is computed as ICC = VC/(VC + 3.29)×100%. The MOR is the median value of the odds ratio between the area of the highest risk and the area of the lowest risk for discriminatory attitudes when two clusters are randomly selected, using clusters as a random variable; MOR = 𝑒0.95√VC. Moreover, the PCV demonstrates the variation in the prevalence of discriminatory attitudes explained by factors. It was computed as $PCV=\frac{Vnull-VC}{Vnull}\times 100\%$ where Vnull = variance of the null model and VC = cluster-level variance. The fixed effects were used to estimate the association between the likelihood of discriminatory attitudes and individual and community-level independent variables. It was assessed, and the strength was presented using an adjusted odds ratio (AOR) and 95% confidence intervals with a *p*-value of < 0.05.

## Result

### Sociodemographic characteristics of the participants

A total of weighted samples of 56,690 women aged 15–49 years were included in this study. More than half, 32,015 (56.47%) of the participants were rural dwellers. The majority 44,936 (79.27%) of the study participants had media exposure. More than two-thirds, 26,168 (68.32%) of the participants faced a big problem in accessing the health institution (Table 2).

**Table 2:** Sociodemographic characteristics of the participants

Individual level variables		Weighted frequency (*n*)	Percentage (%)
Age	15–19 years	11,363	20.04
	20–35 years	29,252	51.60
	36–49 years	16,075	28.36
Educational level	No education	6,692	11.8
	Primary	21,151	37.31
	Secondary	22,959	40.5
	Higher	5,888	10.39
Media exposure	No	11,754	20.73
	Yes	44,936	79.27
Household wealth	Poor	22,987	40.55
	Middle	10,848	19.14
	Rich	22,855	40.32
Marital status	Never married	17,977	31.71
	married	24,415	43.07
	Ever married	14,298	25.22
Currently working	No	28,599	50.45
	Yes	28,091	49.55
Distance to the health facility	Big problem	12,135	31.68
	Not big problem	26,168	68.32
Internet Use	No	36,084	63.65
	Yes	20,606	36.35
Husband education level	No education	5,119	16.05
	Primary	12,475	39.11
	Secondary	9,849	30.87
	Higher	4,457	13.97
Sex of household head	Male	36,327	64.08
	Female	20,363	35.92
Comprehensive knowledge of HIV	No	4,530	72.27
	Yes	1,738	27.73
Ever tested for HIV	No	10,761	25.97
	Yes	30,674	74.03
**Community level variables**			
Residence	Urban	24,675	43.53
	Rural	32,015	56.47
Community media exposure	Low	23,990	42.32
	High	32,700	57.68
Community illiteracy	Low	20,293	35.8
	High	36,397	64.2
Community poverty	Low	22,987	40.55
	High	33,703	59.45
Country category	Gabon	9,400	16.58
	Kenya	32,036	56.51
	Tanzania	15,254	26.91

### Prevalence of discriminatory attitudes against people living with HIV/AIDS in three sub-Saharan African countries

The prevalence of discriminatory attitudes against people living with HIV/AIDS in three sub-Saharan African countries was 28.19% (95% CI: 27.74%, 28.64%).

### Random effect analysis and model fitness

To determine whether the data supported the decision to assess randomness at the community level, a null model was used. Findings from the null model showed that there was a significant variation in discriminatory attitudes between communities, with a variance of 0.6491 and a *p*-value of 0.001. The variance within clusters contributed 83.52% of the variation in discriminatory attitudes, while the variance across clusters was responsible for 16.48% of the variation. In the null model, there is a variation in the odds of discriminatory attitudes between higher and lower risk clusters by a factor of 2.15 times. The intra-class correlation value for model I indicated that 11.39% of the variation in discriminatory attitudes accounts for the differences between communities. Thus, with the null model, to generate model II we have used community-level variables like community media exposure, community poverty, and residence. Cluster variations were the basis for 11.17% of the differences in discriminatory attitudes against people living with HIV/AIDS, according to the ICC value from model II. The final model (model III), attributed approximately 65.21% of the variation in the likelihood of discriminatory attitudes to both individual and community-level variables. The likelihood of discriminatory attitudes varied by 1.81 times across low and high discriminatory attitudes clusters. Moreover, the lowest deviance which was 3481.58 in model III revealed that model III is the best fitted model for data (Table 3).

**Table 3:** Model comparison and random effect analysis for discriminatory attitudes against people living with HIV/AIDS among women of reproductive age in three sub-Saharan African countries

Parameter	Null model	Model I	Model II	Model III
Variance	0.6491	0.4227	0.4139	0.3929
ICC	16.48%	11.39%	11.17%	10.67%
MOR	2.15	1.85	1.84	1.81
PCV	Reference	34.88%	36.23%	65.21%
LLR	-21979.581	-1745.2786	-21705.542	-1740.7894
Deviance	43959.162	3490.5572	43411.084	3481.5788

### Factors associated with discriminatory attitudes against people living with HIV/AIDS among women of reproductive age in three sub-Saharan African countries

In the final fitted model of multivariable logistic regression, the age of the respondent, internet use, comprehensive knowledge about HIV/AIDS, testing for HIV, residence, and community media exposure were factors significantly associated with discriminatory attitudes against people living with HIV/AIDS (Table 4).

The odds of having discriminatory attitudes against people living with HIV/AIDS among women in the age groups of 15–19 and 20–35 years were 1.72 (AOR = 1.72, 95% CI: 1.10, 2.70) and 1.43 (AOR = 1.43, 95% CI: 1.18, 1.74) times higher as compared to those women who were in the age groups of 45–49 years, respectively. The odds of having a discriminatory attitude against people living with HIV/AIDS were 1.39 (AOR = 1.39, 95% CI: 1.12, 1.72) times higher among participants who didn’t use the internet as compared to internet users.

The likelihood of having discriminatory attitudes against people living with HIV/ADIS was 2.26 (AOR = 2.26, 95% CI: 1.81, 2.82) times higher among those individuals who had no comprehensive knowledge as compared to those who had comprehensive knowledge about HIV/AIDS. Having discriminatory attitudes against people living with HIV/AIDS was 1.78 (AOR = 1.78, 95% CI: 1.36, 2.34) times higher among women who didn’t test for HIV as compared to those women who tested for HIV. Having discriminatory attitudes against people living with HIV/AIDS was 1.37 (AOR = 1.37, 95% CI: 1.05, 1.79) times higher among rural dwellers as compared to women who were urban dwellers.

**Table 4:** Multivariable multilevel logistic regression analysis of individual-level and community-level factors associated with discriminatory attitudes against people living with HIV/ADIS among women of reproductive age in three sub-Saharan African countries; DHS 2021/22

Individual and community level variable	Model I AOR (95% CI)	Model II AOR (95% CI)	Model III AOR (95% CI)
Age			
15–19 years	1.74 (1.11, 2.73)		**1.72 (1.10, 2.70)**
20–35 years	1.43 (1.18, 1.74)		**1.43 (1.18, 1.74)**
36–49 years			1
Level of education			
No education	1.55 (0.94, 2.56)		1.57 (0.95, 2.59)
Primary	1.30 (0.84, 2.01)		1.27 (0.82, 1.97)
Secondary	1.06 (0.72, 1.56)		1.05 (0.72, 1.55)
Higher			1
Having media exposure			
Yes	1.18 (0.91, 1.53)		1.17 (0.90, 1.52)
No	1		1
Household wealth			
Poorest	1.21 (0.84, 1.75)		1.10 (0.75, 1.62)
Poorer	1.13 (0.78, 1.63)		1.11 (0.77, 1.60)
Middle	0.84 (0.58, 1.21)		0.83 (0.58, 1.20)
Richer	1.25 (0.88, 1.78)		1.25 (0.88, 1.77)
Richest	1		1
Currently working			
No	1.10 (0.92, 1.32)		1.11 (0.93, 1.33)
Yes	1		1
Distance from health institution			
Big problem	0.97 (0.80, 1.16)		0.95 (0.79, 1.14)
Not big problem	1		1
Internet use			
No	1.40 (1.13, 1.74)		**1.39 (1.12, 1.72)**
Yes	1		1
Husband educational level			
No education	1.15 (0.82, 1.60)		1.16 (0.83, 1.61)
Primary	1.08 (0.72, 1.61)		1.07 (0.72, 1.60)
Secondary	1.10 (0.81, 1.48)		1.10 (0.81, 1.49)
Higher	1		1
Gender of the household head			
Male	1.09 (0.89, 1.34)		1.08 (0.88, 1.33)
Female	1		1
Comprehensive knowledge about HIV/AIDS			
No	2.26 (1.81, 2.83)		**2.26 (1.81, 2.82)**
Yes	1		1
Tested for HIV			
No	1.79 (1.37, 2.35)		**1.78 (1.36, 2.34)**
Yes	1		1
Residence			
Rural		1.40 (1.32, 1.49)	**1.37 (1.05, 1.79)**
Urban		1	1
Community media exposure			
High		0.77 (0.69, 0.86)	1.18 (0.84, 1.65.)
Low		1	1
Community illiteracy level			
High		0.46 (0.41, 0.51)	1.00 (0.78, 1.28)
Low			1

## Discussion

Our study aimed to assess discriminatory attitudes against people living with HIV/AIDS and associated factors in three sub-Saharan African countries. A finding of this study revealed that the prevalence of discriminatory attitudes against people living with HIV/AIDS was 28.19% (95% CI: 27.74%, 28.64%). This finding is lower than the prior studies done in Ethiopia [24, 29], sub-Saharan African countries [25], Zambia [26], Botswana [27], and Saudi Arabia [28]. The possible reason for this variation might be due to socio-cultural variation, study period, and sample size.

This study also identified factors associated with discriminatory attitudes against people living with HIV/AIDS. The odds of discriminatory attitudes against people living with HIV/AIDS were higher among women in the age groups of 15–19 and 20–35 years as compared to those women who were in the age group of 45–49 years. This finding is supported by a prior study [25]. This could be because of the variation in their level of maturity and life experiences. Additionally, those older women may have an opportunity to access health-related education and share their experiences at the workplace.

Being an internet user was associated with lower odds of discriminatory attitudes against people living with HIV/AIDS. The media have played a central role in providing information about HIV/AIDS [10]. It could be because people who were internet users may have had an opportunity to access health-related and updated information about HIV/AIDS that minimizes the preexisting myths and harmful attitudes against HIV/AIDS.

Having a comprehensive knowledge of HIV/AIDS was associated with lower odds of discriminatory attitudes against people living with HIV/AIDS. This is in agreement with the previous studies [25, 29, 31–34]. This might be because having knowledge regarding HIV transmission and myths regarding HIV/AIDS minimizes stigmatization and discrimination.

Moreover, a finding of this study revealed that testing for HIV was associated with lower odds of discriminatory attitudes against people living with HIV/AIDS. This is consistent with the previous studies [29, 30, 34]. It could be because of pre-test education and post-test counseling on the basic concepts of HIV testing and counseling. It is anticipated that this could provide a chance to avoid myths regarding HIV/AIDS. Additionally, clients undergoing HIV testing may receive information about the various interventions that are available, which is crucial to dispelling myths like discrimination.

We found that the odds of discriminatory attitudes against people living with HIV/AIDS were higher among rural dwellers as compared with those who were urban dwellers. This finding is consistent with the previous study findings [25, 29, 30, 33–35]. It could be because, in urban areas, it may be easier to access accurate information about HIV/AIDS, internet service, formal and informal HIV/AIDS education, and a comprehensive knowledge of HIV transmission and prevention mechanisms. Thus, the prevalence of HIV-related discrimination declines.

As a strength of the study, it uses nationally representative data, a large sample size, and appropriate statistical analysis. As a limitation, important factors that could have a great impact on discriminatory attitudes against people living with HIV/AIDS, like behavior, beliefs, and social norms, are not included in the dataset. Additionally, to measure the discriminatory attitudes against people living with HIV/AIDS, a social desirability bias may have been present in the women’s verbal responses. These will prevent the intended impact of our findings. Thus, further studies should be carried out to explore women’s discriminatory attitudes against people living with HIV/AIDS by considering the issues mentioned above.

## Conclusion

The prevalence of discriminatory attitudes against people living with HIV/AIDS in three sub-Saharan African countries was high. Individual and community-level variables were associated with discriminatory attitudes against people living with HIV/AIDS. Therefore, special consideration should be given to rural dwellers and young adults. In addition, better to strengthen the accessibility of Internet and HIV testing services, and improve HIV-related education to reduce the magnitude of discriminatory attitudes against people living with HIV/AIDS.

### Electronic supplementary material

Below is the link to the electronic supplementary material.

Supplementary Material 1

## Data Availability

The datasets generated and/or analyzed during the current study are available in the most recent data of the Demographic and Health Survey and it is publicly available online at (https://www.dhsprogram.com).
