# Supplementary material for: Individual and community level factors associated with discriminatory attitudes against people living with HIV/AIDS among women of reproductive age in three sub-Saharan African countries: evidence from the most recent demographic and health survey (2021/22)
Source: BMC Public Health. 2024 Jun 5;24:1503. doi: 10.1186/s12889-024-19022-7 (PMC11151550; doi:10.1186/s12889-024-19022-7)
Supplement: Supplementary file 1 — Supplementary Material 1 [file 12889_2024_19022_MOESM1_ESM.pdf]

STATA (version 14) commands

Null model: melogit Discrimination [iw=wt]||v001:,or,

Model I: melogit Discrimination ib3.age_category ib3.v106 ib0.Media_exposure ib5.v190 ib1.v714 ib2.v467d ib1.Internet ib3.Husband_education ib2.v151 ib1.knowledge_HIV ib1.v781 [iw=wt]||v001:,or,

Model II: melogit Discrimination ib1.v025 ib0.Community_media ib0.Community_education [iw=wt]||v001:,or,

Model III: melogit Discrimination ib3.age_category ib3.v106 ib0.Media_exposure ib5.v190 ib1.v714 ib2.v467d ib1.Internet ib3.Husband_education ib2.v151 ib1.knowledge_HIV ib1.v781 ib1.v025 ib0.Community_media ib0.Community_education [iw=wt]||v001:,or,
